# A dataset of EEG and ECG recordings around the onset of NREM sleep from infancy to adolescence

**DOI:** 10.1038/s41597-026-07356-3

**Published:** 2026-05-05

**Authors:** Nathan J. Stevenson, Kartik K. Iyer, James A. Roberts, Eero Ahtola, Leena Lauronen, Sampsa Vanhatalo

**Affiliations:** 1 Brain Modelling Group, QIMR Berghofer, Brisbane, Australia; 2 Clinical Neurophysiology, New Children’s Hospital, Helsinki University Hospital and University of Helsinki, Helsinki, Finland; 3 Paediatric Research Center, New Children’s Hospital, Helsinki University Hospital, Helsinki, Finland; 4 BABA Center, Department of Physiology, University of Helsinki, Helsinki, Finland

## Abstract

Paediatric brain activity can be measured effectively during light sleep; a vigilance state that manifests with similar phenomenology on the EEG across childhood. Here, we describe a curated dataset of EEG and ECG recordings from 1032 subjects from 2 months to 16 years of age (Helsinki Kids 1 K – HK1K). All subjects had age-appropriate EEG and ECG recordings, along with typical neurodevelopment, as confirmed by a clinical review of their medical records over the four years following the recording. These data can be used to define normative ranges of paediatric EEG/ECG, train foundation models of paediatric EEG/ECG, and generate age prediction algorithms that underpin measures of brain age gap.

## Background & Summary

Electroencephalography (EEG) is routinely used for assessing cortical neuronal activity in the clinical and research contexts. It is well known that several visually salient characteristics of the EEG change after birth, most prominently during the first years of life. Any assessment of paediatric EEG recordings, therefore, needs to consider the child’s age as a benchmark.

There is considerable interest in measuring brain age as a surrogate biomarker of health in paediatric populations. The difference between a child’s chronological age and brain age assessed from the EEG^1,2^, structural MRI data^3^ or, more indirectly, via measurement of autonomic function from the ECG^4,5^, is believed to provide an objective measure of neurodevelopment. However, much of the literature on brain age measures has been dominated by structural imaging in adult populations^6^. Brain age predictions based on functional measurements (e.g. EEG, fMRI, fNIRS) trained and validated on paediatric populations, where age is an established, fundamental benchmark for a range of clinical assessments (https://www.who.int/tools/child-growth-standards/standards), are relatively scarce^1,7–10^.

Paediatric EEG measurements provide a widely scalable resource for building efficient computational tools as they are performed routinely for clinical purposes worldwide. International guidelines have ensured reasonable harmonization of recording methodology, including standardized electrode placements, requirements for EEG hardware, and minimum specifications of amplifier characteristics, such as sampling frequency and filters^11,12^. Beyond the technical harmonization of EEG recordings, it is well known that a child’s vigilance level affects many characteristics in waking EEG activity, such as the frequency of posterior dominant rhythm (reactive alpha rhythm). Recording EEG activity at a standard level of waking vigilance across childhood is not feasible in practice. It is, however, possible to obtain a standard of vigilance by selecting epochs around the time of N2 sleep onset, which have clearly defined onset criteria that are approximately uniform from early infancy to adolescence^11^.

We have previously developed age prediction algorithms using a large cohort of routinely collected EEG and ECG measurements from over 1000 Finnish children during sleep^1,5^. This data descriptor outlines the dataset used in these studies, with slight modifications, which are available on Zenodo^13^. Our dataset consists of EEG and ECG recordings with segments visually extracted from around the onset of N2 sleep in a large cohort of infants, children and adolescents with no abnormalities on EEG review and no neurological diagnosis from birth until 4 years after the recording. This is a unique, publicly available dataset of EEG and/or ECG recordings that is technically and behaviourally harmonized across the age span and complementary to existing paediatric sleep datasets^14^. Our dataset (Helsinki Kids 1 K – HK1K) is open access and provides a large-scale resource to expand training datasets, improve the generalizability of predictors across multi-centred cohorts, generate pre-trained feature extractors and support external validation.

## Methods

### Data acquisition

The dataset was initially compiled as part of a study into the brain activity of infants, children and adolescents around the onset of N2 sleep^1^. Data collection began with a retrospective screening of hospital records at the paediatric clinical neurophysiology unit of the Helsinki University Central Hospital from 2011 to 2016 resulting in an initial cohort of 1056 recordings. All EEG reports were reviewed through the 5-year period, and the present dataset was collated using three inclusion criteria: First, the clinical EEG report by the board-certified EEG expert was normal. Second, there were no medications that are known to potentially affect brain activity. Third, the child’s neurodevelopment was typical as defined by absent neurology-related diagnoses within four years of the EEG recording (see^1^ for more details). This is a reliable method for excluding neurological diagnoses, because all children with neurology-related disorders in the Finnish health care system are systematically referred to specialist paediatric clinics in the same hospital consortium (including child neurology and child psychiatry) for diagnosis and care.

**Fig. 1:**
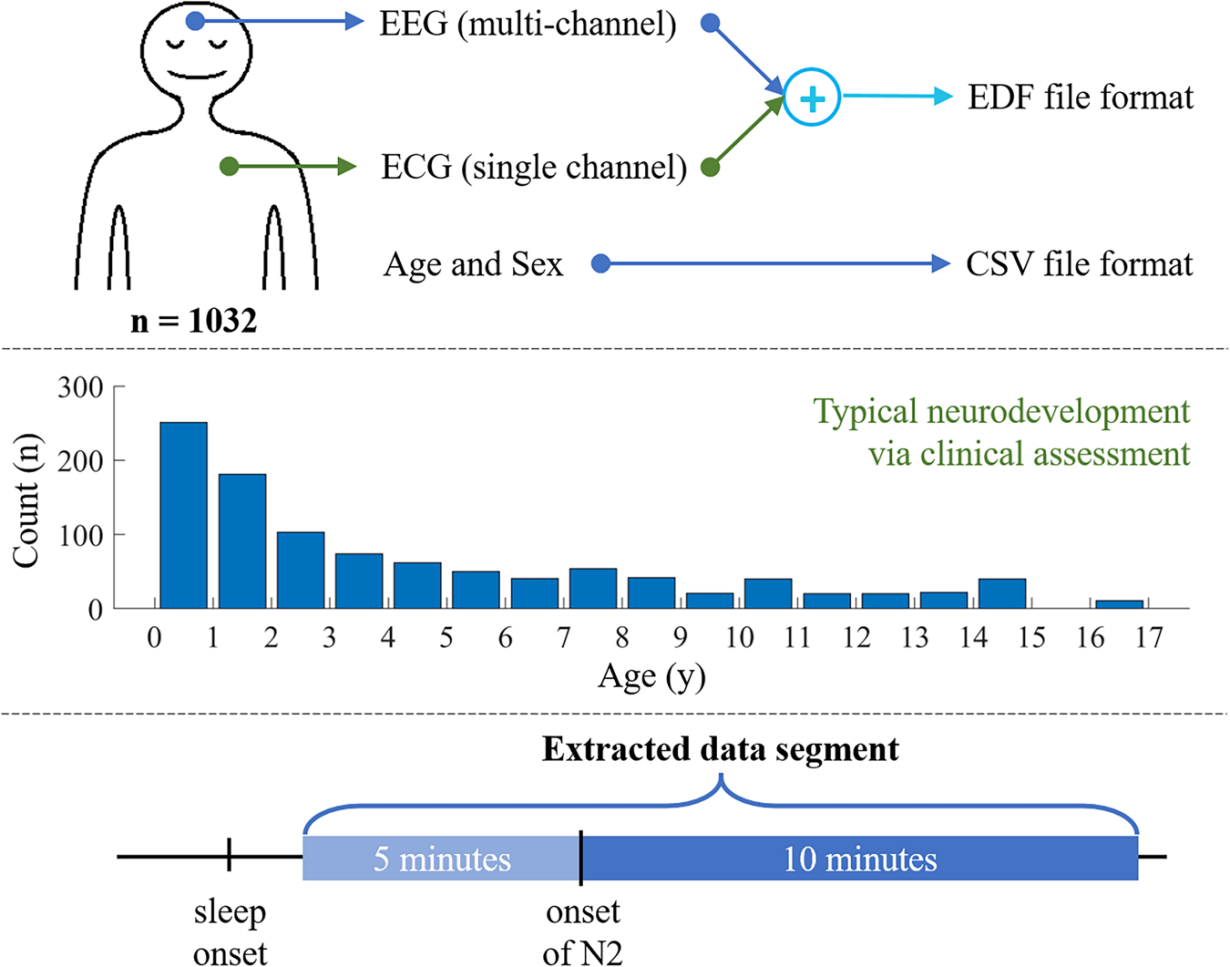
Design of data collection protocol.

These routine EEG studies of children (0–16 years) included a single-channel ECG, and the measurement session contained periods of both wake and sleep, with the aim of collecting at least 10 min of N2 sleep as identified by an EEG technician with special training in sleep scoring and over 30 years’ experience in paediatric EEG and sleep studies (see Fig. 1).

Helsinki University Hospital and its paediatric units comprise a network of tertiary level hospitals, which provide a comprehensive paediatric neurology service for 1.8 million people around the Helsinki metropolitan area. EEG studies are requested for clinical indications by the treating child neurologist, paediatrician or child psychiatrist, and nearly all EEG recordings are performed as an out-patient service in the clinical neurophysiology lab of each paediatric hospital campus. The night before the EEG study, parents are instructed to reduce their child’s sleeping time by waking up their child two to four hours earlier than their normal wake-up time to ensure sufficient sleep pressure is present before entering the laboratory.

In this dataset, we extracted EEG and ECG signals from all available measurements. The EEG was recorded using an EEG cap (Waveguard, ANT Neuro, Hengelo, Netherlands) connected to a NicoletOne EEG system (Natus Medical Inc. Middleton, WI, USA). All signals were acquired with a common sampling frequency of 250 Hz. The recordings used the same 19 EEG electrodes at standard 10–20 locations (Fp1, Fp2, F7, F3, Fz, F4, F8, T3/T7, C3, Cz, C4, T4/T8, T5, P3, Pz, P4, T6, O1, and O2). Clinical EEG review was typically performed with a longitudinal bipolar montage (“double banana”; Fp1-F7, F7-T3, T3-T5, T5-O1, Fp1-F3, F3-C3, C3-P3, P3-O1, Fp2-F2, F4-C4, C4-P4, P4-O2, Fp2-F8, F8-T4, T4-T6, T6-O2, Fz-Cz, and Cz-Pz). Two ECG electrodes were placed on the chest and recorded as an additional bipolar channel, also sampled at 250 Hz. Several other polygraphic channels could be recorded using age-dependent and patient-dependent variations, such as respiratory effort, two EMG channels, or eye movements with a piezoelectric sensor; however, these channels were excluded from our dataset.

**Fig. 2:**
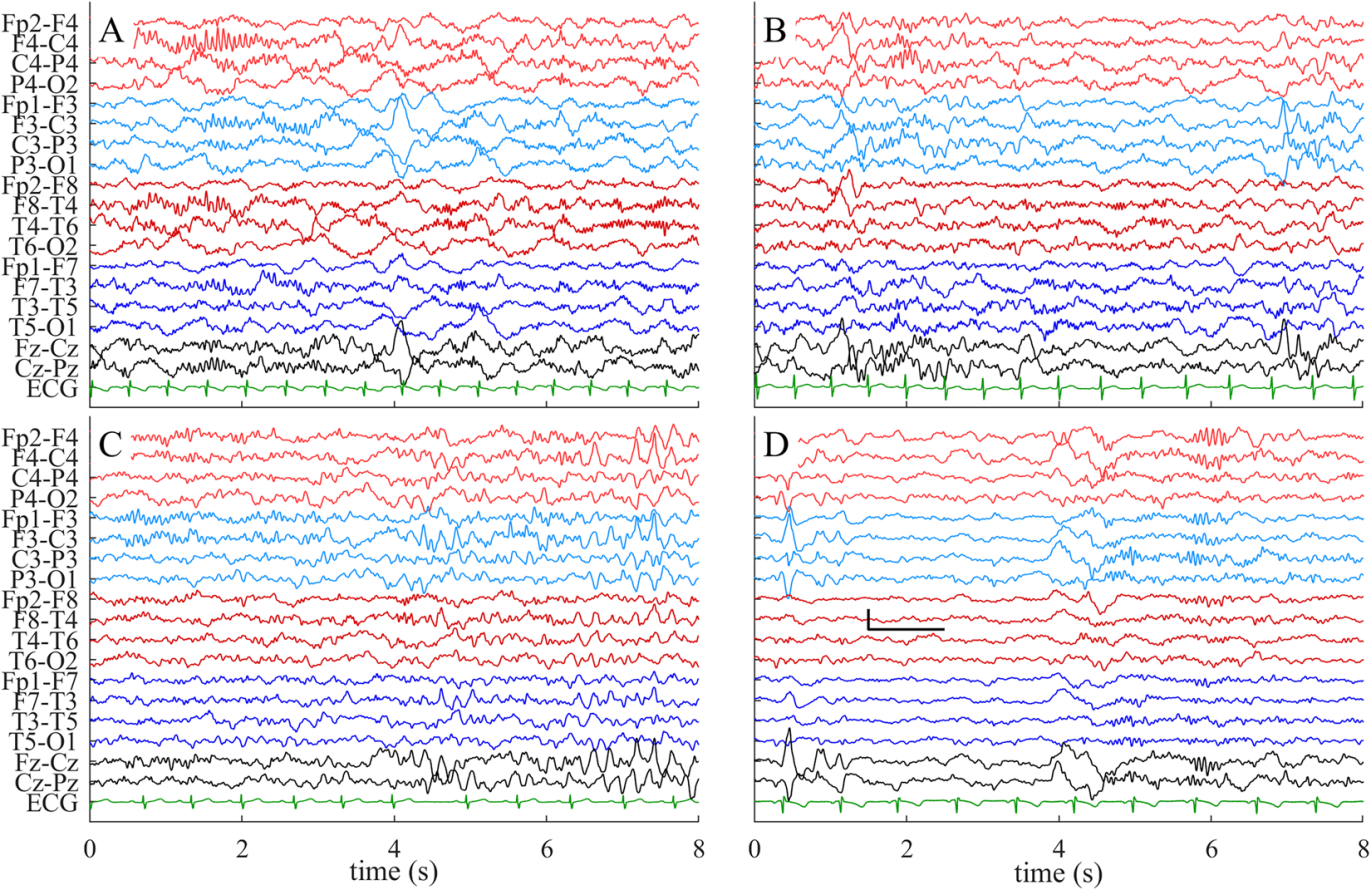
Exemplar EEG/ECG recordings from the dataset around the transition to N2 sleep. (**A**) 9 months of age, (**B**) 3 years of age, (**C**) 5 years of age, and (**D**) 13 years of age. The distance between labels on the y-axis represents 100 µV; a graticule is shown in (**D**) with a vertical range of 100 µV and a horizontal range of 1 s for visual reference - the same scale is used in all plots.

A 15-minute segment was extracted from each recording (see Fig. 2 for exemplar epochs). The segment was defined in relation to the onset of N2 sleep (5 minutes before and 10 minutes after), defined by the first occurrence of sleep spindles or K-complexes in EEG recordings (according to AASM guidelines^15^; and using a standard longitudinal montage – see Fig. 1). Although sleep architecture is immature in early infancy, the key EEG grapho-elements used to define N2 sleep—most notably sleep spindles—are clearly identifiable and routinely used in paediatric EEG interpretations throughout the age range in our cohort, from 2 months to 16 years of age and onwards^11,16^.

The initial dataset was reduced to simplify prospective processing of the data. A small subset of recordings was contaminated with runs of zeros associated with fragmentation due to impedance checks or changes in acquisition settings – these recordings were included in our original study^1^ but required additional processing steps during analysis. This subset was, therefore, removed (n = 24) resulting in 1032 recordings from 1032 subjects.

As the priority for the present dataset collection was EEG signal quality, there was no additional criteria related to ECG quality, i.e., several poor-quality ECG recordings were included (we define poor as being unable to use the ECG recording to calculate an accurate heart rate signal via R peak detection methods; approximately 28 recordings – see^5^). Demographics within this dataset are limited with no information on socio-economic status, although the sampled population was primarily of white/Eurasian descent.

Data collection was approved by the Institutional Research Review Board at Helsinki and the New Children’s Hospital (HUS/244/2021), including waiver of consent due to secondary use (science) of retrospectively collected data that was initially collected as standard of care. The public release was approved after our data anonymization protocol was formally approved by the Finnish social and health data permit authority (FinData; https://findata.fi/en/). In other words, FinData determined that the EEG/ECG recordings were non-identifiable (anonymized) due to the following criteria: all recording-specific metadata was removed from the EEG and ECG signals (including references to date and time, annotations, any personal details) and over-written with a generic header to preserve file structure; all files were renamed with random codes and the code-book (connecting random code to original EDF filenames) was permanently discarded; the child’s age was quantized/discretized so that every discrete age value included at least 10 children.

### Data anonymization

All information relating to dates, names, and patient IDs were removed from file headers and file names of each recording. Recording names were assigned a random code that was linked to demographic variables (age and sex) and recorded in a single codex for initial testing. The random seed to generate the codex was itself selected randomly and the codex linking recording ID to random ID has since been deleted from the computer used to perform the assignment.

Age was discretized so that at least 10 subjects were assigned at each age level. As the data were collected with a positive skew in the distribution with age, age discretization was nonlinear/nonuniform and the assignment of ages into bins of 10 subjects per bin began from the oldest subjects and worked down to the youngest subjects (see Code at https://github.com/nstevensonUH/EEG_release_supporting_code). Discretization was, therefore, data driven with a bin size that increases, approximately, logarithmically with age. Example segments of the EEG/ECG recordings are shown in Fig. 2.

## Data Records

The dataset is freely available to access at Zenodo^13^. The dataset contains the following files:

- **EDF files** (edf_files.zip) containing 19 EEG channels in a referential montage (Fp1-Ref, Fp2-Ref, F7-Ref, F3-Ref, Fz-Ref, F4-Ref, F8-Ref, T3/T7-Ref, C3-Ref, Cz-Ref, C4-Ref, T4/T8-Ref, T5-Ref, P3-Ref, Pz-Ref, P4-Ref, T6-Ref, O1-Ref, and O2-Ref) and one ECG channel (ECG1-ECG2) from 1032 subjects with an age range of 2 months to 16 years of age sampled at 250 Hz. EEG and ECG units are microvolts (µV). Files are stored with a unique randomly generated 6-character filename (e.g. 2Y32V8.edf).
- **One CSV file** (anon_codex_age_sex.csv) that contains clinical data (columns: quantized age and quantized sex) acquired from patient notes aligned with each randomly generated EDF filename (column: Anon. Filename). Age is non-uniformly discretized and defined in years and sex is defined as either male (Male) or female (Female).

## Technical Validation

An initial visual inspection was performed to ensure EEG activity was present in the majority of recordings (in duration and across channels). The visual assessment was supported by quantitative EEG (qEEG) analyses including measures of EEG amplitude and spectral power. Amplitude was used as a surrogate measure of contamination, while spectral power served as a measure of overall recording quality.

EEG may be distorted by various sources of artefact – several of these, such as movement or poor electrode contact, are associated with considerable increases in EEG amplitude above typical values. We calculated the number of seconds EEG amplitude exceeded 500 µV in a recording (with a resolution of 8 s – this value ensures that EEG activity around a potential high amplitude artefact is highlighted during additional processing and as such our measures constitute an overestimate). The median duration of recording affected by high amplitude was 0.38% (IQR: 0.06% to 1.49%; min = 0.0%, max = 22.1%; n = 1032).

**Fig. 3:**
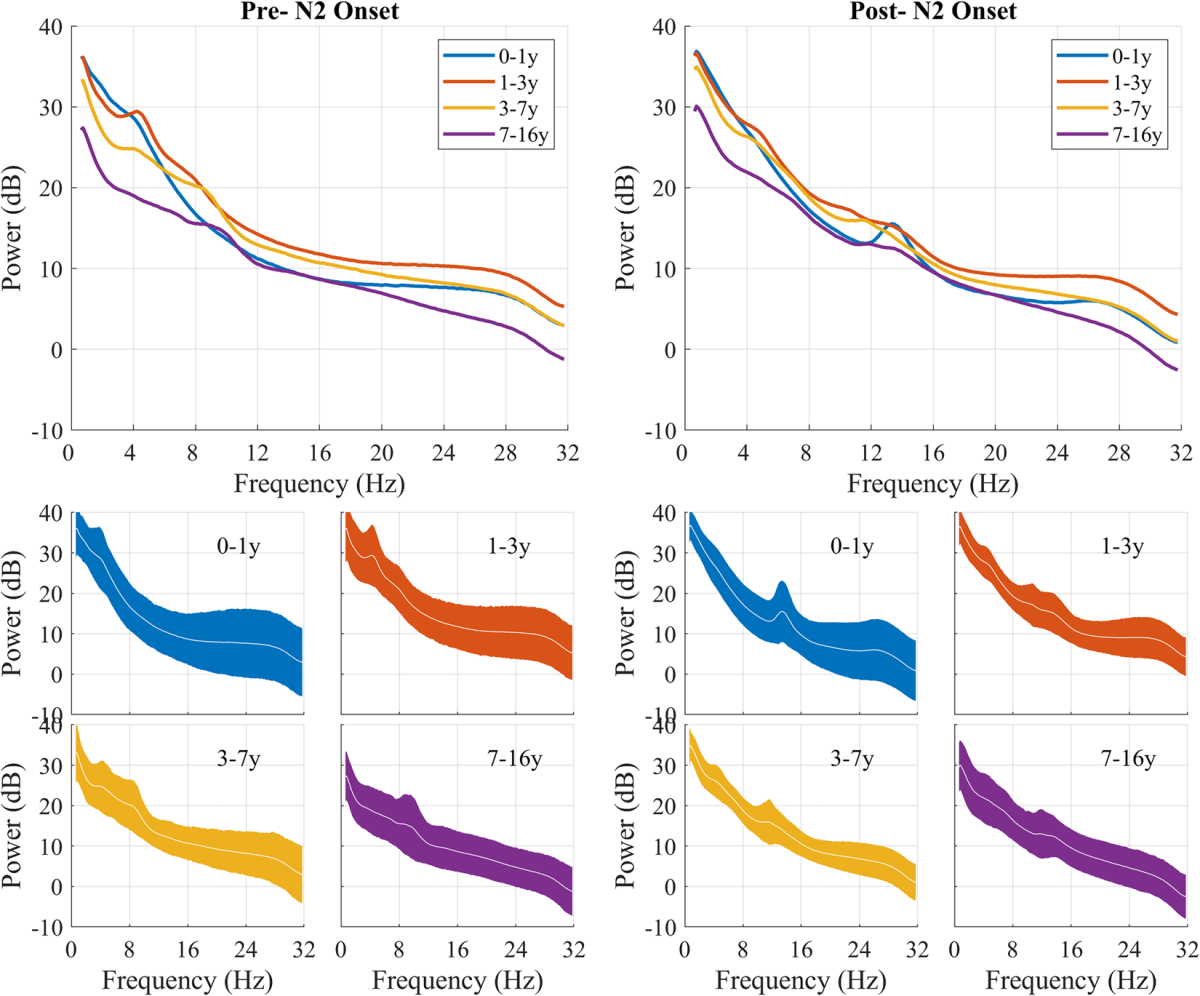
Power spectral densities of EEG averaged across all recording channels in a longitudinal bipolar montage of the data. The PSDs separated into four age ranges, with 95% confidence intervals are shown below each summary plot – the white line is the mean PSD from the combined PSD plot at the top of this figure.

The quality of EEG recordings was assessed by comparing low frequency activity (0.5–16 Hz) to high frequency activity (70–125 Hz). The power spectral density (PSD) of each EEG recording was used to calculate band powers (periodogram, Fs = 250 Hz, 63 signal segments were used per recording – including a 50% overlap – and signal segments were zero padded to a length of 8192 samples, see Fig. 3). Spectral band power calculations were performed per EEG channel in a bipolar montage (excluding midline derivations Fz-Cz and Cz-Pz) and then averaged across channels. The median high frequency band power was 0.10 μV^2^/Hz (IQR: 0.04 – 0.24; min = 0.004, max = 5.90; n = 1032) and the median low frequency power was 275.9 μV^2^/Hz (IQR: 151.2–423.1; min = 12.3, max = 1477.8; n = 1032). Both high and low frequency band power were associated with age (t-statistic = 2.04, p < 0.05 and t-statistic = −31.52, p < 0.001, respectively) but not sex (t-statistic = −0.62, p = 0.53 and t-statistic = 0.14, p = 0.88, respectively) in a linear regression model.

The quality of non-overlapping 5-minute epochs from each ECG recording was assessed using spectral analysis, quality detection, and the ECG-derived tachogram. After applying a notch filter at 50 and 100 Hz and a high-pass filter (all filters were 4^th^ order Butterworth and the high-pass filter cutoff was 0.5 Hz), the PSD of each ECG recording were divided into four bands representing movement artifact (0.5–1 Hz), heart rate (1–3 Hz), cardiac waveform elements and harmonics (3–25 Hz), and high frequency background noise (25–125 Hz). The 0.5–1 Hz power was 0.5 μV^2^/Hz (IQR: 0.2–0.9; min = 0.003, max = 79.9; n = 3057 non-overlapping 5-minute epochs), the 1–3 Hz band power was 7.1 mV^2^/Hz (IQR: 2.9–14.3; min = 0.7, max = 1692.5; n = 3057), the median cardiac waveform band power was 11.3 mV^2^/Hz (IQR: 6.0–20.8; min = 0.1, max = 737.0), and the high frequency band power was 0.6 mV^2^/Hz (IQR: 0.2–1.4; min = 0.01, max = 153.1). In addition, quality assessment was performed on each non-overlapping 5-minute epoch of ECG. Epochs were deemed to be poor quality based on the analysis of nine ECG features (total spectral power, maximum auto-correlation, relative spectral power in 6 bands [0–1, 1–3, 3–10, 10–20, 20–60, 60–125] Hz, and spectral entropy) by a support vector machine (see ref. ^5^ for more details). The number of non-overlapping 5-minute ECG epochs deemed as poor quality was 124 (out of 3057 epochs). Finally, a modified Pan-Tompkins algorithm was applied to the raw ECG trace, after passing the previous quality assessment, to determine heart rate over time. The median number and duration of periods within a 5-minute epoch where the peak of the R wave could not be found due to contamination (defined using ECG amplitude) was 0 (95%CI: 0 - 3, min = 0, max = 7) and 0 seconds (95%CI: 0 – 15 s; min = 0 s, max = 62 s).

**Fig. 4:**
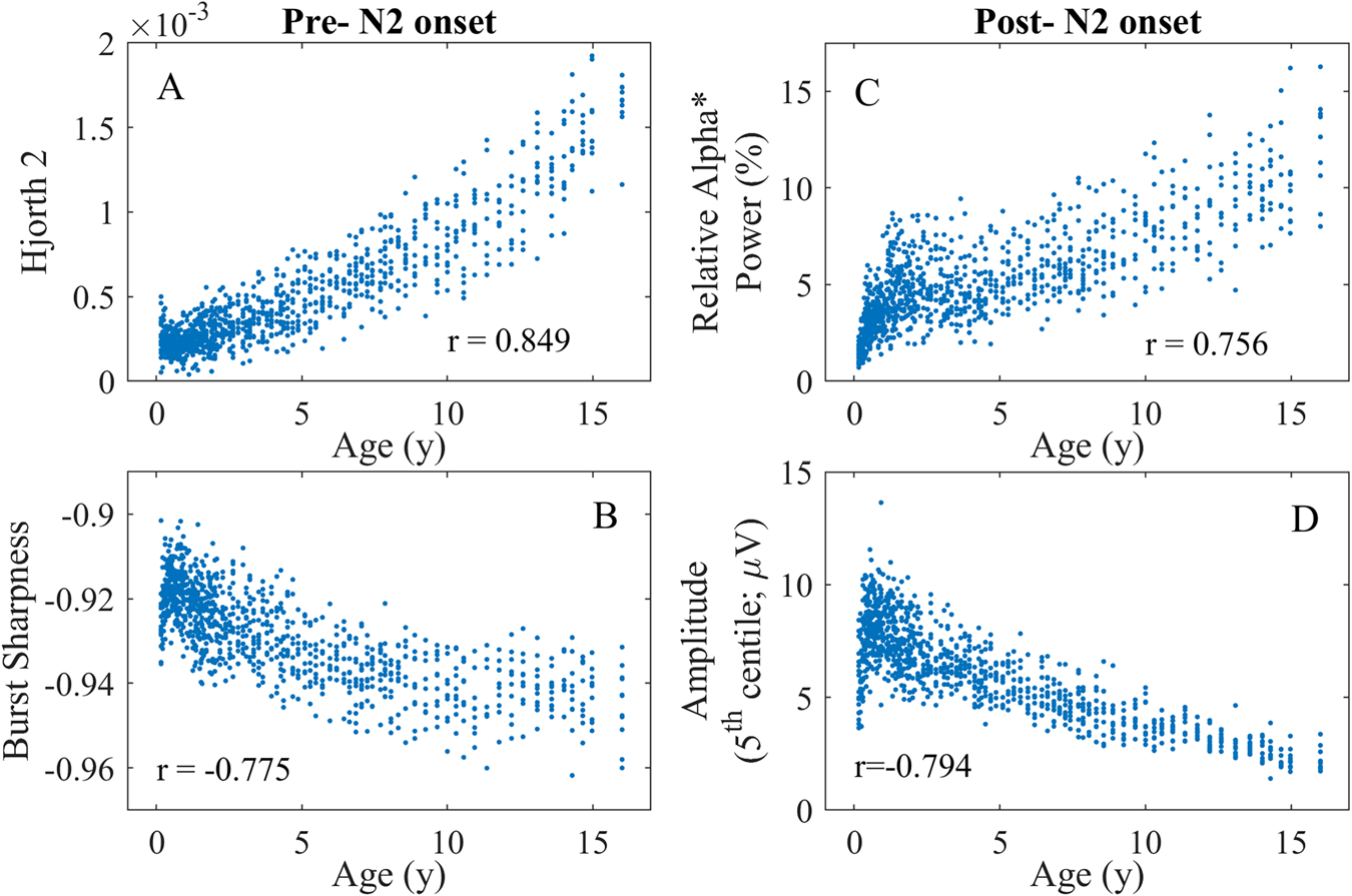
The relationship between exemplar quantitative EEG features and age. (**A**) Global average of mobility (Hjorth parameter 2) calculated on the period of EEG pre-N2 onset. (**B**) Global average of burst sharpness (all bursts) calculated on the period of EEG pre-N2 onset. (**C**) Global average of relative alpha power (* alpha was defined as 8–16 Hz in this case to preserve systematic scaling in the frequency bands and a constant bandwidth across age) calculated on the period of EEG post-N2 onset. (**D**) Global average of the 5^th^ centile of EEG amplitude calculated on the period of EEG post-N2 onset. All calculations were performed in a bipolar EEG montage (longitudinal, double banana); the global average was, therefore, applied to features extracted from multiple 30 s epochs and 18 channels. Outliers were removed for display purposes, but were included in the calculation of Spearman’s correlation coefficient, r.

**Fig. 5:**
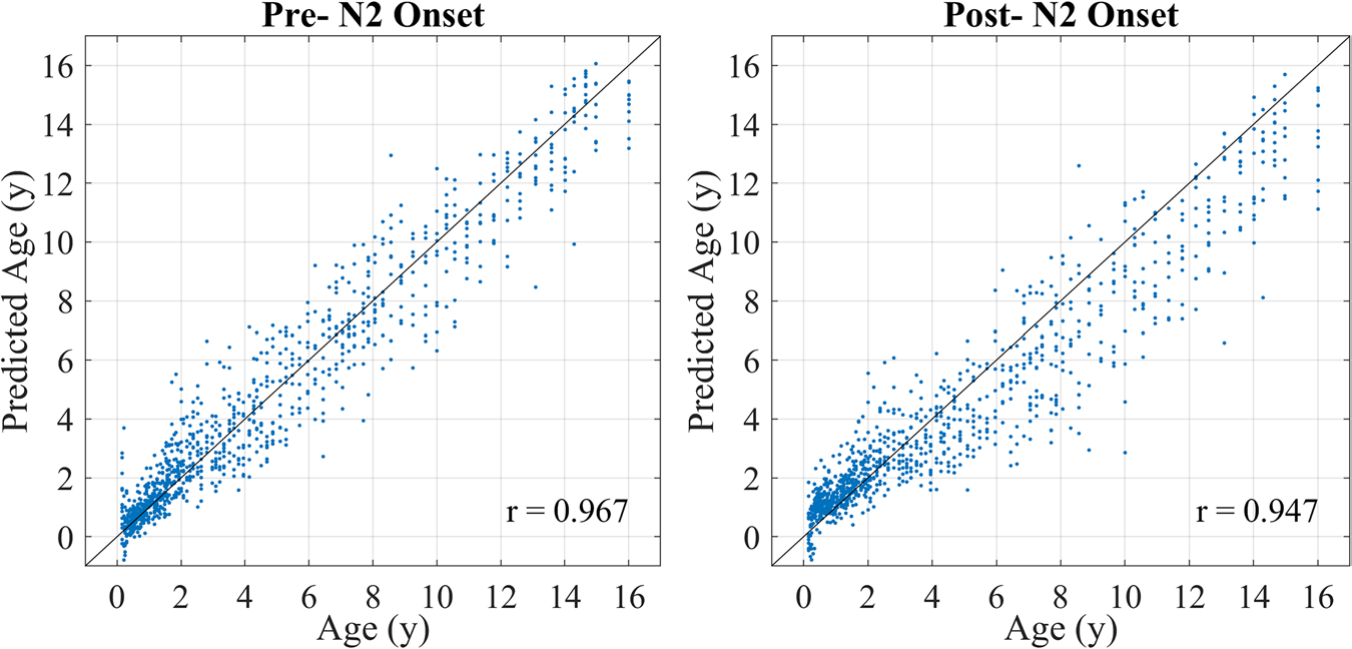
Age prediction from the EEG based on qEEG features combined via Gaussian process regression (GPR). Age predictions were calculated within a 5-fold cross-validation and GPR parameters included standardization, maternal 5/2 kernel and a constant basis function. The mean absolute error was 0.8 years for data recorded pre-N2 onset and 1.1 years for data recorded post-N2 onset. The Pearson’s correlation coefficient (r) between predicted age and age is shown within the figure. Note, conservative feature-based outlier removal resulted in a cohort of n = 1002.

**Fig. 6:**
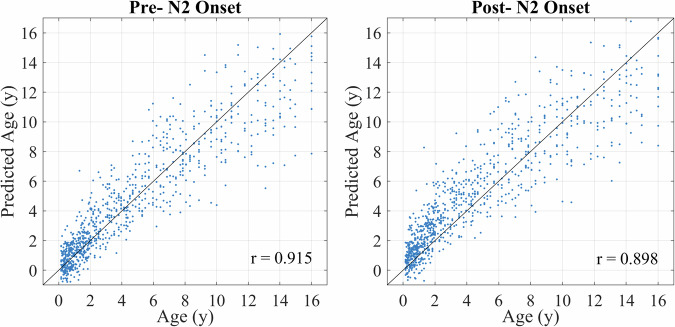
Age prediction from the ECG based on HRV features combined via Gaussian process regression (GPR). Age predictions were calculated within a 5-fold cross-validation and GPR parameters included standardization, maternal 5/2 kernel and a constant basis function. The mean absolute error was 1.2 years for data recorded pre-N2 onset and 1.4 years for data recorded post-N2 onset.  The Pearson’s correlation coefficient (r) between predicted age and age is shown within the figure. Note, conservative feature-based outlier removal resulted in a cohort of n = 843.

We also tested the representation of age within the dataset using qEEG features - the qEEG feature set from^1^ was used. Exemplar relationships between qEEG features and age are shown in Fig. 4 based on recording averages of 30 s epochs of data. The combination of these features forms an accurate prediction of age (see Fig. 5). Similarly, a prediction of age from the ECG-derived tachogram, based on 5 minute epochs of data, was also generated using several features of heart rate variability (see Fig. 6). For all age predictions, data were removed if an initial assessment of the feature value corrected for age exceeded 6 standard deviations.

## Usage Notes

We have tested opening the EDF files using the following EEG viewers: EDF Browser (https://www.teuniz.net/edfbrowser/; version 2.13), Nicolet EDF Viewer/Reader (version 5.94.1.534), BESA Research (version 7.1), and StratusEEG Review (version 5.1). We have successfully imported EDF files into Matlab using our code available from Github (https://github.com/nstevensonUH/EEG_release_supporting_code), EEGLab (https://sccn.ucsd.edu/eeglab/index.php; version 2021.0, with BIOSIG plugin) and SPM Software Package (https://www.fil.ion.ucl.ac.uk/spm/; spm_25.01.02). We have also successfully imported EDF files into Python (version 3.12.9) using MNE (https://martinos.org/mne/stable/index.html.

There is some variability in the location of the reference due to the practicalities of clinical data acquisition. The reference can be identified in each recording as the channel with, essentially, zero EEG amplitude (e.g. if Fz-Ref = 0, then the reference was Fz).

This cohort was derived from clinically referred children and many referrals in paediatric EEG practice arise from common and largely benign concerns. All EEGs were reported as normal by board-certified experts and typical neurodevelopment was confirmed through longitudinal clinical follow-up, providing a stringent clinical definition of neurotypicality. As with any large dataset, a small number of false negatives (children with unrecognized conditions) cannot be entirely excluded. However, paediatric medical care in Finland is accessible and comprehensive, including programmed, population-wide medical assessments throughout childhood, making it unlikely that a child with significant issues would go undiagnosed through the four-year follow-up period used in our cohort. We, therefore, assume that the cohort closely approximates a population-relevant normative reference suitable for developmental, neurophysiological and/or sleep research.

While anchoring analyses to the AASM-defined onset of N2 sleep provides a robust and widely accepted standard of vigilance across childhood, more subtle developmental changes in sleep-onset physiology (e.g. spectral power or amplitude characteristics not explicitly used in N2 scoring) may nonetheless contribute to age-related physiological trends. In addition, all recordings were obtained under a standardized clinical protocol that includes partial sleep deprivation (i.e. early wake-up that morning), to increase the likelihood of obtaining a brief epoch of sleep during the day. It is possible that sleep deprivation (or sleeping during the day) may influence some physiological features at N2 onset in an age-dependent manner, but this is an unavoidable aspect of our data collection protocol. These considerations are common to paediatric neurophysiological research and underscore the importance of applying comparable acquisition and epoch-selection protocols when using this dataset as a normative reference.

## Data Availability

The dataset is freely available to access at Zenodo (10.5281/zenodo.17138539).

## Code availability

MATLAB code that assists in accessing the EEG and ECG recordings within this dataset, and reproduces all analyses undertaken in this data descriptor, is available on Github (https://github.com/nstevensonUH/EEG_release_supporting_code).
